# Opioid-related overdose deaths among people experiencing homelessness, 2017 to 2021: A population-based analysis using coroner and health administrative data from Ontario, Canada

**DOI:** 10.23889/ijpds.v9i5.2492

**Published:** 2024-09-18

**Authors:** Richard Booth, Salimah Shariff, Brooke Carter, Stephen Hwang, Aaron Orkin, Cheryl Forchuk, Tara Gomes

**Affiliations:** 1 Arthur Labatt Family School of Nursing, Western University; 2 ICES; 3 London Health Sciences Centre Research Institute; 4 MAP Centre for Urban Health Solutions, Li Ka Shing Knowledge Institute, Unity Health Toronto; 5 Department of Family and Community Medicine, University of Toronto; 6 Dalla Lana School of Public Health, University of Toronto; 7 Leslie Dan Faculty of Pharmacy, University of Toronto

## Background

We aimed to measure the change in proportion of opioid-related overdose deaths attributed to people experiencing homelessness and to compare the opioid-related fatalities between individuals experiencing homelessness and not experiencing homelessness at time of death.

## Approach

We conducted a population-based, time-trend analysis using coroner and health administrative databases from Ontario, Canada (2017-2021). Quarterly proportion of opioid-related overdose deaths attributed to people experiencing homelessness were reported. We also obtained socio-demographic and health characteristics, healthcare encounters preceding death, substances directly contributing to death and circumstances surrounding deaths. 

## Results

Overall, 13.3% individuals who died of an opioid-related overdose between 2017 and 2021 were identified as experiencing homelessness at the time of death. The quarterly proportion of opioid-related overdose deaths attributed to people experiencing homelessness increased from 7.2% in 2017 to 16.8% by 2021. Compared with housed decedents, those experiencing homelessness were younger, had higher prevalence of mental health or substance use disorders and more often visited hospitals and emergency departments in the year prior to death. Fentanyl and stimulants more often directly contributed to death, whereas methadone was less often present. Individuals experiencing homelessness were more often in the presence of a bystander during the event that led to death and more often had a resuscitation attempted or naloxone administered. 

## Conclusion

People experiencing homelessness account for an increasing proportion of fatal opioid-related overdoses in Ontario, Canada, reaching nearly one in six such deaths in 2021. Effective interventions are needed to combat homelessness and opioid related toxicity in homeless populations.

